# Structural Mechanisms Determining Inhibition of the Collagen Receptor DDR1 by Selective and Multi-Targeted Type II Kinase Inhibitors

**DOI:** 10.1016/j.jmb.2014.04.014

**Published:** 2014-06-26

**Authors:** Peter Canning, Li Tan, Kiki Chu, Sam W. Lee, Nathanael S. Gray, Alex N. Bullock

**Affiliations:** 1 Structural Genomics Consortium, University of Oxford, Old Road Campus, Roosevelt Drive, Oxford OX3 7DQ, UK; 2 Department of Cancer Biology, Dana Farber Cancer Institute, Boston, MA 02115, USA; 3 Department of Biological Chemistry and Molecular Pharmacology, Harvard Medical School, Boston, MA 02115, USA; 4 Cutaneous Biology Research Center, Massachusetts General Hospital and Harvard Medical School, Charlestown, MA 02129, USA

**Keywords:** A-loop, activation loop, CML, chronic myeloid leukemia, DDR, discoidin domain receptor, IGF1R, insulin-like growth factor 1 receptor, ITC, isothermal titration calorimetry, KID, kinase insert domain, RTKs, receptor tyrosine kinases, TCEP, tris(2-carboxyethyl)phosphine, TKI, tyrosine kinase inhibitor, TrkB, tropomyosin-related kinase B, phosphorylation, crystallography, drug design, oncology, gleevec

## Abstract

The discoidin domain receptors (DDRs), DDR1 and DDR2, form a unique subfamily of receptor tyrosine kinases that are activated by the binding of triple-helical collagen. Excessive signaling by DDR1 and DDR2 has been linked to the progression of various human diseases, including fibrosis, atherosclerosis and cancer. We report the inhibition of these unusual receptor tyrosine kinases by the multi-targeted cancer drugs imatinib and ponatinib, as well as the selective type II inhibitor DDR1-IN-1. Ponatinib is identified as the more potent molecule, which inhibits DDR1 and DDR2 with an IC_50_ of 9 nM. Co-crystal structures of human DDR1 reveal a DFG-out conformation (DFG, Asp-Phe-Gly) of the kinase domain that is stabilized by an unusual salt bridge between the activation loop and αD helix. Differences to Abelson kinase (ABL) are observed in the DDR1 P-loop, where a β-hairpin replaces the cage-like structure of ABL. P-loop residues in DDR1 that confer drug resistance in ABL are therefore accommodated outside the ATP pocket. Whereas imatinib and ponatinib bind potently to both the DDR and ABL kinases, the hydrophobic interactions of the ABL P-loop appear poorly satisfied by DDR1-IN-1 suggesting a structural basis for its DDR1 selectivity. Such inhibitors may have applications in clinical indications of DDR1 and DDR2 overexpression or mutation, including lung cancer.

## Highlights

• DDR kinases bind potently to clinically relevant type II kinase inhibitors.
• Co-crystal structures of DDR1 reveal the inhibitor binding modes.
• Structural differences to other kinases allow for DDR-selective inhibitors.
• Type II kinase inhibitors have potential to treat DDR-associated diseases.

## Graphical Abstract

**Figure:**
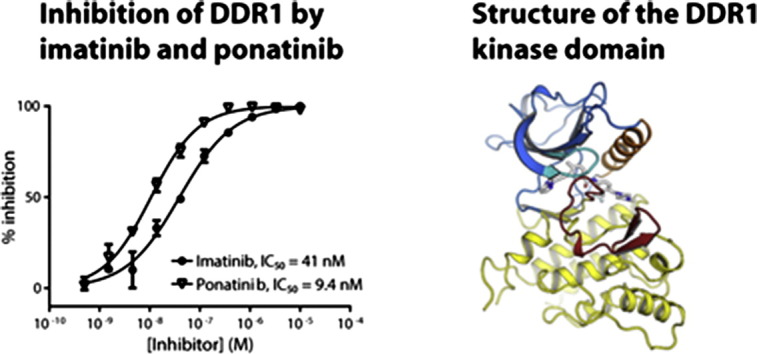

## Introduction

The discoidin domain receptors (DDRs), DDR1 and DDR2, are unique among the receptor tyrosine kinases (RTKs) in being activated by interaction with the extracellular matrix [1,2]. Binding to triple-helical collagen is mediated by the receptor extracellular domains that include an N-terminal discoidin (DS) domain, a DS-like domain and a short juxtamembrane (JM) region [3–5]. A single transmembrane helix links to the cytoplasmic domain, where a larger JM region precedes the catalytic C-terminal kinase domain. Both DDRs form constitutive dimers making them unusual among RTKs, which typically dimerize only upon activation [6–8]. DDRs regulate extracellular matrix remodeling, as well as cell adhesion, proliferation and migration [9]. DDR1 is expressed mainly in epithelial cells where it plays an important role in mammary gland development [10], whereas mesenchymal expression of DDR2 promotes bone growth, as suggested by dwarfism in DDR2 knockout mice [11].

DDR kinases are linked to the progression of various human diseases, including fibrotic disorders, atherosclerosis and cancer [9,12,13]. Significantly, they are identified as indicators of poor prognosis in ovarian, breast and lung cancer [14–16]. DDR1 overexpression is associated with increased cell survival and invasion in hepatocellular carcinomas, pituitary adenoma and prostate cancer [17–19], whereas DDR2 is mutated in squamous cell lung cancers [20] and contributes to breast cancer metastasis [21]. The promise of DDR kinases as a therapeutic target has been demonstrated by DDR1 knockdown that has been shown to reduce metastatic activity in lung cancer models [22], slow the development of atherosclerosis [13] and impede the development of fibrotic disorders [23–25].

Imatinib (STI-571) is a first-line tyrosine kinase inhibitor (TKI) targeted at breakpoint cluster region-Abelson kinase (ABL) for the treatment of chronic myeloid leukemia (CML) [26]. As a type II inhibitor, imatinib achieves significant selectivity by binding to an inactive DFG-out conformation (DFG, Asp-Phe-Gly) of the kinase domain [27]. A chemical proteomics study recently identified DDR1 as a secondary target of imatinib, leading to the suggestion that DDR1 inhibition may also contribute to the effectiveness of the treatment [28], particularly as activation of DDR1 is known to block p53-mediated apoptosis [29]. Further characterization of this interaction revealed imatinib to be a potent inhibitor of DDR1, as were the second-generation TKIs nilotinib and dasatinib [30]. Moreover, dasatinib may have potential to treat squamous cell lung cancer in patients harboring oncogenic mutations in DDR2 [20]. Imatinib also rescues mouse models of fibrosis [31,32] similarly to DDR1 deficiency [25], although a connection between these effects has yet to be proven. Ponatinib is a third-generation TKI developed for the treatment of CML patients with resistance to imatinib [33,34]. It was selected primarily to circumvent the steric hindrance introduced by the ABL T315I “gatekeeper” mutation and has proven to be a more potent but considerably less selective inhibitor than imatinib [30]. Finally, the inhibitor DDR1-IN-1 was designed to a similar pharmacophore model as these multi-targeted type II kinase inhibitors but has been recently reported as a highly selective pharmacological probe for DDR1-dependent signal transduction [35]. Such inhibitors will be highly valuable to investigate further the complex roles of DDR1 in both normal and pathobiology. In addition, more selective compounds are likely to offer improved safety profiles for potential clinical indications outside oncology.

While crystal structures of DDR1 and DDR2 have revealed the molecular basis for extracellular collagen interaction [5,36], a structural description of the kinase domain fold is lacking. Here, we present the crystal structures of the kinase domain of human DDR1 in complexes with the inhibitors imatinib and ponatinib, as well as structural comparisons to the selective inhibitor DDR1-IN-1. The structures reveal differences to ABL in both the shape and the sequence of the ATP pocket that can be exploited for the design of DDR1-specific inhibitors.

## Results

### Structure determination

**Fig. 1:**
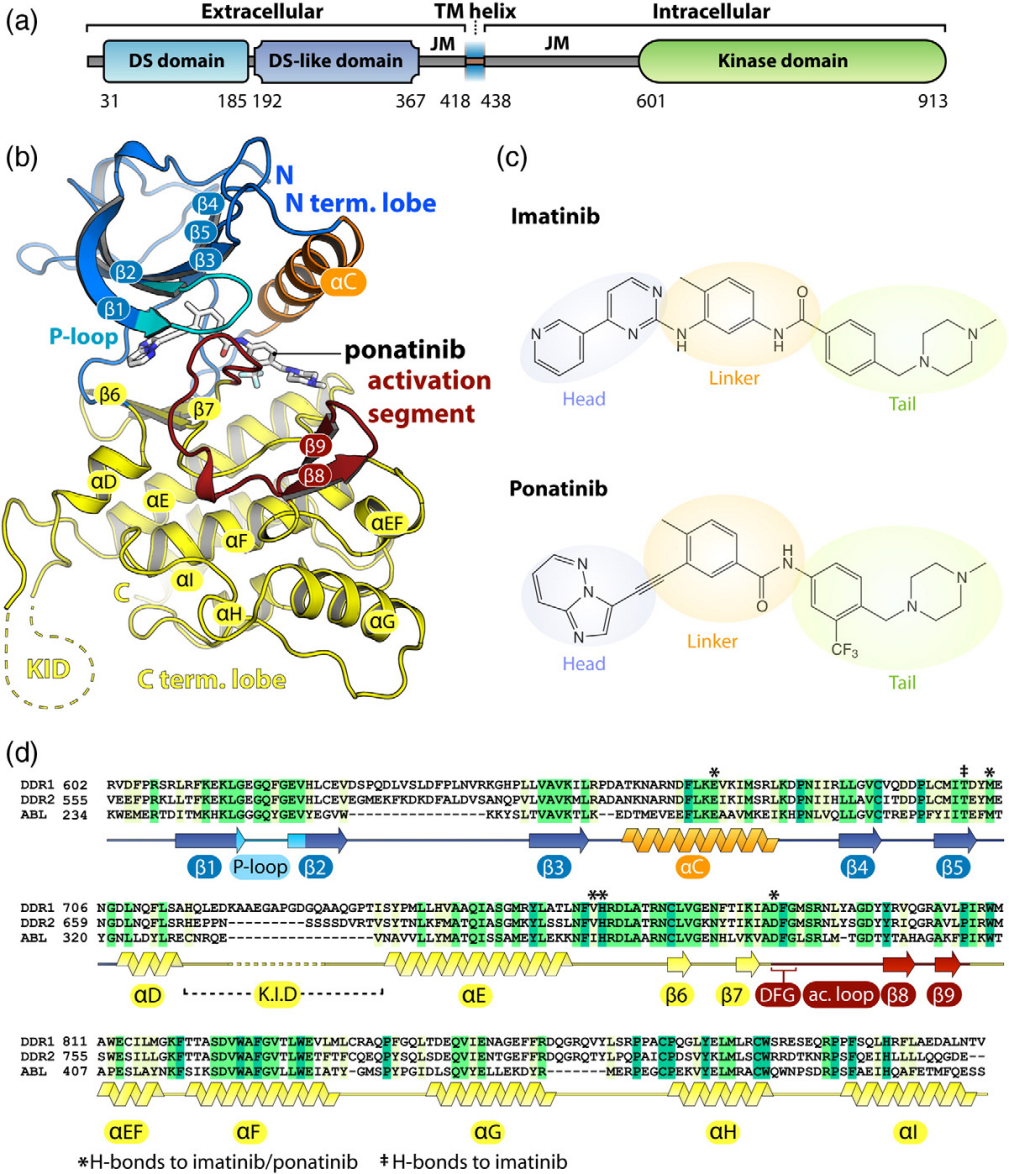
Overview of the DDR1 structure. (a) Domain organization of DDR1. (b) Crystal structure of the DDR1 kinase domain in complex with the inhibitor ponatinib. (c) Chemical structures of imatinib and ponatinib, highlighting their “head”, “linker” and “tail” regions. (d) Sequence alignment of the kinase domains of DDR1, DDR2 and ABL. Secondary structure elements are displayed for the DDR1 kinase. Residues labeled with an asterisk (*) form hydrogen bonds with both imatinib and ponatinib. Residues labeled with a double dagger (^‡^) form hydrogen bonds with imatinib only.

**Fig. 2:**
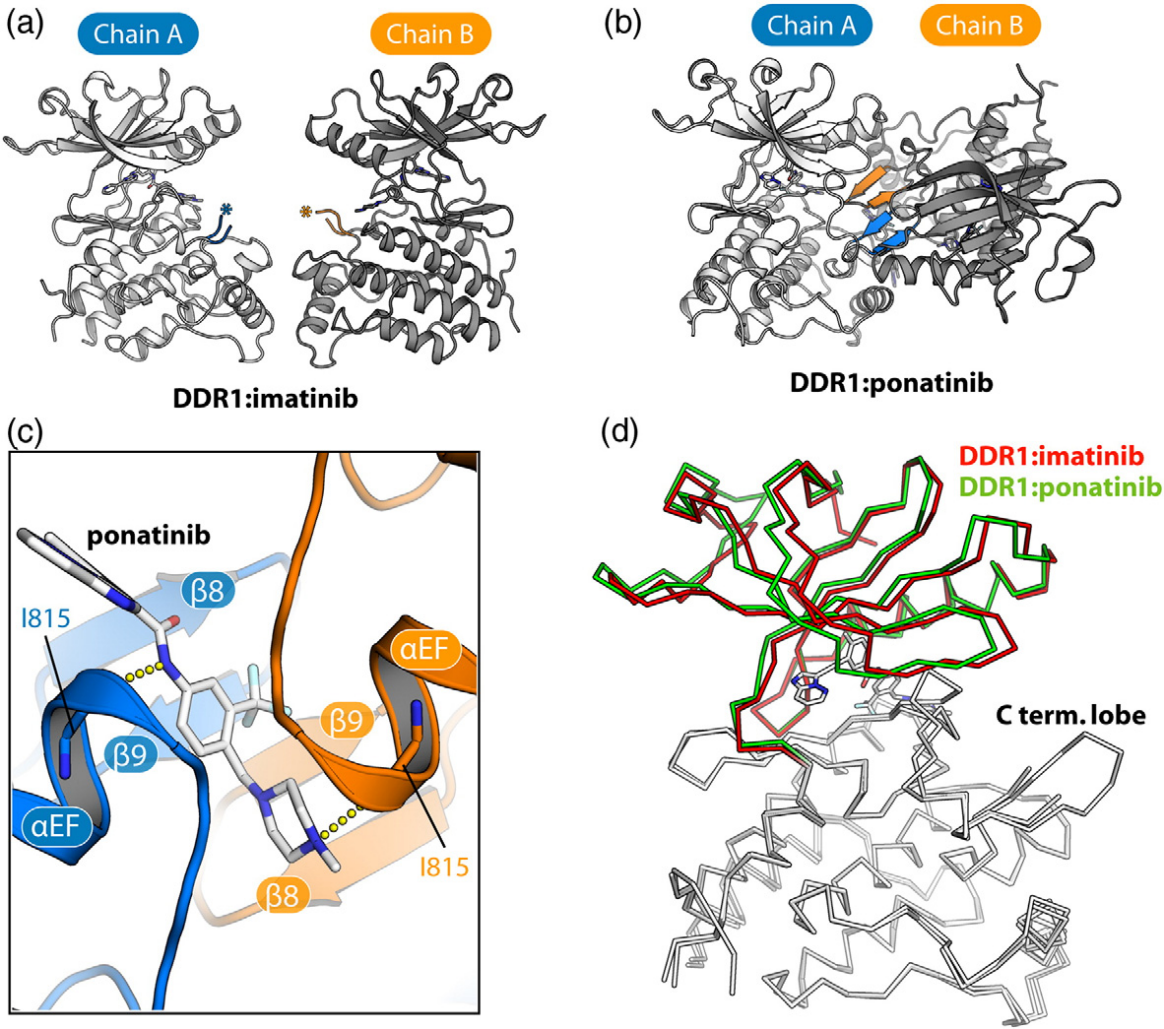
Crystal packing in the DDR1–ponatinib complex. (a) The asymmetric unit of the DDR1–imatinib structure contains two protein monomers. Asterisks mark the disordered regions of the activation segments corresponding to strands β8 and β9. (b) The asymmetric unit of the DDR1–ponatinib structure contains a crystallographic dimer held by the self-association of the β8-β9 hairpin (colored blue and orange in chains A and B, respectively). (c) The dimer interface is stabilized by the binding of an additional ponatinib molecule. The linker and tail regions of the interfacial ponatinib molecule contact hydrophobic residues on the lower face, where they also hydrogen bond to the carbonyl of Ile815 (αEF helix) in each protein chain. The opposite face of the β-sheet is stabilized by contacts with the αC helix and phosphate-binding loop (P-loop), including a salt bridge between Arg798 (β8) and Asp668 (αC) (data not shown). (d) Perhaps as a result of these different packing interactions, DDR1 monomers from the imatinib and ponatinib co-structures show a subtle shift in the relative positions of the N-terminal kinase lobes.

The kinase domain of human DDR1 (residues 601–913; Fig. 1a) was expressed in Sf9 insect cells and purified using nickel affinity and size-exclusion chromatography. Crystal structures (Fig. 1b) were determined in separate complexes with the kinase inhibitors imatinib and ponatinib, respectively (Fig. 1c). The ponatinib co-structure was solved by molecular replacement using tropomyosin-related kinase B (TrkB) (PDB ID: 4AT5) [37] as a search model and refined at 1.9 Å resolution (see Table 1 for data collection and refinement statistics). The complete main chain was traceable, except for residues 721–731, which were not visible in the electron density map. This site, corresponding to a small kinase insert domain (KID), was one of several sequence insertions identified relative to the comparable kinase domain structure of ABL (Fig. 1d). The DDR1–imatinib complex structure was solved subsequently using data collected from crystals improved by microseeding procedures (see Materials and Methods). The structure was solved by molecular replacement and refined to 1.7 Å resolution. Disordered regions in the imatinib co-structure were identified in both the KID region and a portion of the activation segment spanning residues 799–803. The two inhibitor complexes exhibited distinct crystal packing leading to small differences in their respective structures (Fig. 2). DDR1 was monomeric in the imatinib complex, consistent with its size-exclusion profile, whereas the ponatinib complex formed a crystallographic dimer with an additional ponatinib molecule bound at the dimer interface (Fig. 2).

Overall, DDR1 displays the classical bilobal architecture of a tyrosine kinase (Fig. 1b). An N-terminal extension folds across the top of the smaller N-terminal lobe, which comprises β1–β5 strands and the αC helix. In the α-helical C-terminal lobe, the activation segment includes an additional β-hairpin motif formed by strands β8 and β9 (Fig. 1b and d). As observed for ABL, both type II inhibitors induce an inactive conformation of DDR1 characterized by a “DFG-Asp out, αC-Glu in” configuration. In this conformation, the catalytically relevant salt bridge is observed between DDR1 residues Glu672 (αC) and Lys655 (β3), but the remaining catalytic site is disrupted by an inverted conformation of the DFG motif in the activation loop (A-loop).

### The DDR family fold

**Fig. 3:**
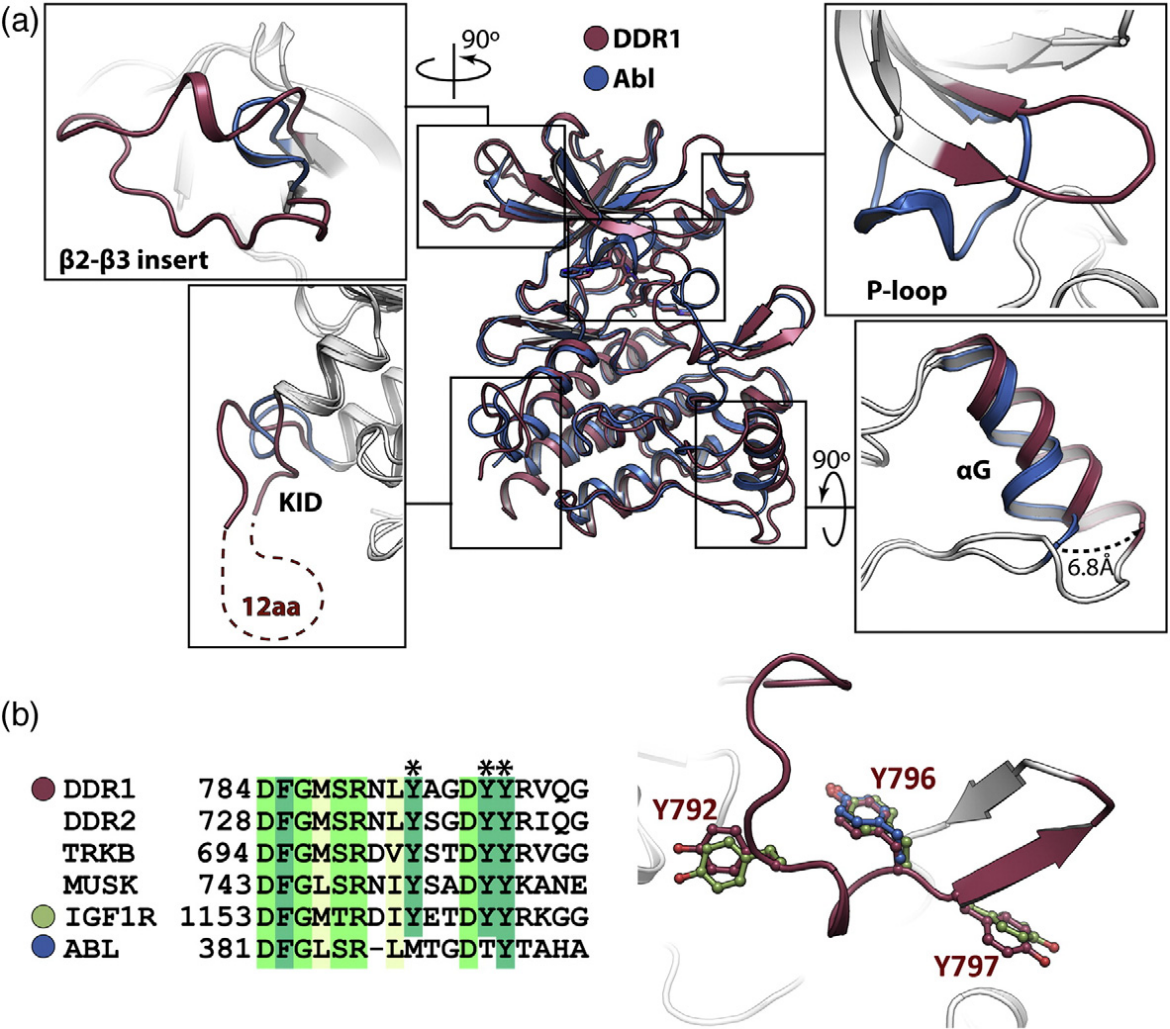
Structural comparison of DDR1 and ABL. (a) Superposition of the structures of DDR1 and ABL–imatinib (PDB ID: 2HYY) [38] reveals a number of structural changes that are highlighted by boxed regions. (b) Sequence alignment of the activation segment of selected kinases. The three potential sites of phosphorylation in DDR1 are highlighted by asterisks. The same region is highlighted on the DDR1 structure (right) showing the same tyrosine side chains in DDR1 (red with residue numbers displayed), IGF1R (PDB ID: 1P4O; green) [39] and ABL (blue) [38].

Comparable inhibitor-bound structures of DDR1 and ABL show a global root-mean-square deviation (RMSD) of 3.6 Å over 248 C^α^ atoms (Fig. 3a), with identical values for the respective imatinib and ponatinib complexes. DDR1 contains a notable insertion between the β2 and β3 strands, where amino acids 629–650 form a structured loop of 22 residues that is double the length of the same region in the closely related kinases insulin-like growth factor 1 receptor (IGF1R) [40], MUSK (muscle, skeletal receptor tyrosine protein kinase) [41] and TrkB [37]. These residues fold across the top of the N-lobe forming hydrophobic interactions with the N-terminal extension and β5 strand. The N-lobes also differ in the P-loop, which in DDR1 remains bound to β3 as a β-hairpin in contrast to the cage-like structure of ABL that dissociates to envelope the bound imatinib (Fig. 3a). Two additional sequence insertions are identified in the C-lobe. Firstly, DDR1 contains a small KID located between helices αD and αE, although 12 of these 23 residues are disordered (Fig. 3a). Secondly, an insert of 7 residues follows the DDR1 αG helix leading to a difference of 6.8 Å in the positions of the DDR1 and ABL chains at this site (Fig. 3a).

Similar insertions are predicted in DDR2, although the KID region appears 9 residues shorter in this protein (Fig. 1d). The two DDR family members share 68% sequence identity within their kinase domains, with the highest conservation as expected in the catalytic core and activation segments. In particular, the P-loop and ATP pocket residues of DDR1 and DDR2 are strictly conserved suggesting that their inhibitor binding preferences will be similar. Both kinases contain three potential sites of tyrosine phosphorylation in their A-loops corresponding to DDR1 Tyr792, Tyr796 and Tyr797. Their packing closely matches that of the conserved residues in the inactive structures of IGF1R [40], MUSK [41] and TrkB [37] (Fig. 3b). In particular, DDR1 Tyr796 adopts a conserved position as a pseudosubstrate, as observed also for the single tyrosine in ABL [38] (Fig. 3b). Mass spectrometry confirmed that the DDR1 kinase domain was expressed and purified in a non-phosphorylated state.

### Interactions of the P-loop

**Fig. 4:**
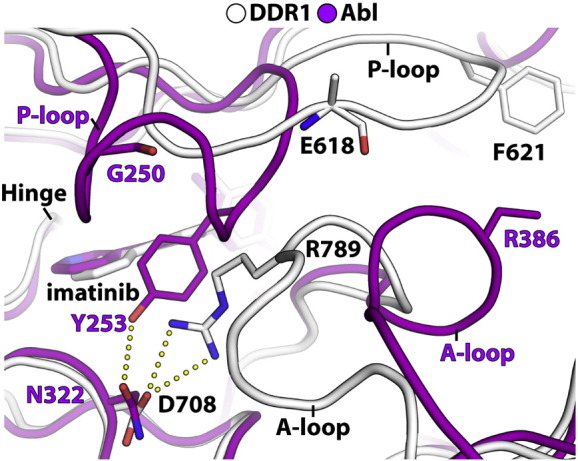
Divergent P-loop structures in DDR1 and ABL lead to changes in ATP pocket shape. Superposition of the DDR1–imatinib (white) and ABL–imatinib (PDB ID: 2HYY; magenta) [38] complexes. DDR1 Glu618 and Phe621 are equivalent to the drug resistance mutations Gly250Glu and Tyr253Phe in ABL. Different conformations are also observed for the A-loop where DDR1 Arg789 corresponds to ABL Arg386.

Perhaps the most important sequence changes in the DDR family occur in the P-loop, where ABL mutations conferring imatinib resistance (Gly250Glu and Tyr253Phe) match the native DDR sequence (DDR1 Glu618 and Phe621, respectively). In ABL, Tyr253 forms a hydrogen bond with Asn322 to stabilize the cage-like structure of the P-loop, while Gly250 likely contributes to the flexibility required for this fold (Fig. 4). This P-loop conformation also affords additional van der Waals interactions with the inhibitor. As a result of its phenylalanine substitution, the same hydrogen bond cannot be formed in DDR1 and its P-loop retains the common β-hairpin conformation. Interestingly, the missing P-loop interactions are replaced by the A-loop, which inserts DDR1 Arg789 into the ATP pocket to hydrogen bond with the Asn322-equivalent residue Asp708 (Fig. 4). This conformation is further stabilized by main-chain hydrogen bonding between the P-loop and A-loop (data not shown). Thus, the alternative structure of DDR1 removes Glu618 and Phe621 from the binding site into solvent while maintaining suitable ATP pocket interactions for inhibitor binding.

### Binding of imatinib and ponatinib

**Fig. 5:**
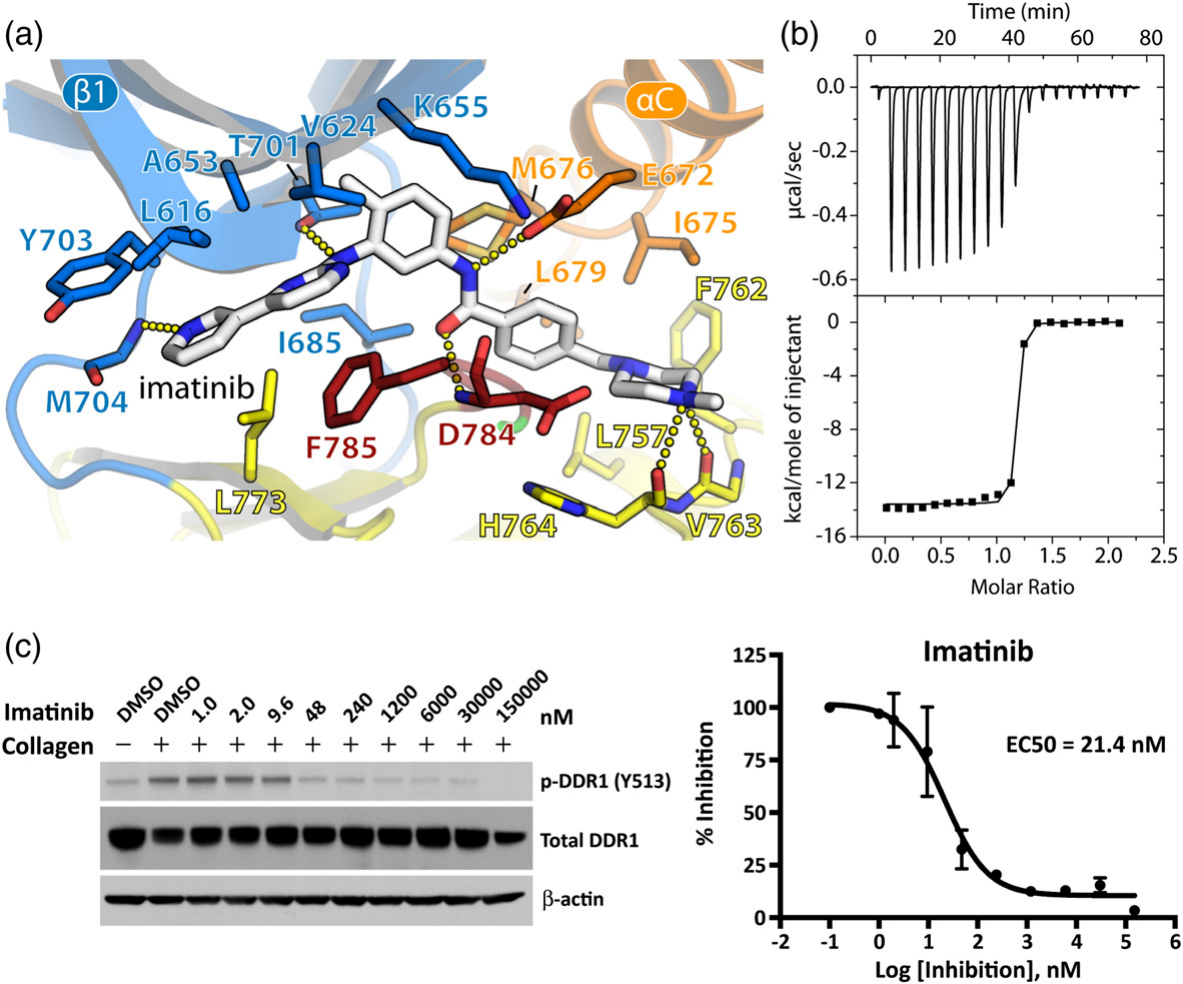
DDR1 binding and inhibition by imatinib. (a) Interactions of imatinib in the ATP pocket of DDR1. Colors correspond to the structural features indicated in Fig. 1. Hydrogen bond interactions are shown as dotted lines. (b) ITC measurements of the binding show a *K*_D_ value of 1.9 nM. (c) Imatinib efficacy on blocking collagen-induced DDR1 Y513 autophosphorylation in U2OS cells.

As anticipated, the binding of both type II inhibitors is facilitated by an inverted conformation of the DFG motif that exposes an additional binding pocket below the αC for the inhibitor tail, while the head groups bind to the hinge. In total, imatinib forms six hydrogen bonds in the ATP pocket of DDR1 (Fig. 5a). Two target the hinge region, including one between the pyridine head group and the backbone amide of Met704 and one between the aminopyrimidine and the gatekeeper residue Thr701. Two more are made by the linker: the amide binds to Glu672 in the αC, while the carbonyl contacts the backbone amide of Asp784 in the DFG motif. Finally, the methylpiperazine group occupies a hydrophobic pocket between the αC and HRD (His-Arg-Asp) motif and hydrogen bonds with the backbone carbonyls of Val763 and His764. The DFG motif further stabilizes the binding with a π–π stacking interaction between Phe785 and the aminopyrimidine group (Fig. 5a). The strong binding of DDR1 to imatinib was revealed by isothermal titration calorimetry (ITC), which indicated a dissociation constant (*K*_D_) of 1.9 nM (Fig. 5b). To confirm the ability of imatinib to inhibit DDR1 kinase activity in cells, we measured its ability to block collagen-induced DDR1 autophosphorylation in U2OS cells. Again, imatinib demonstrated significant potency against DDR1 yielding an EC_50_ of 21 nM.

**Fig. 6:**
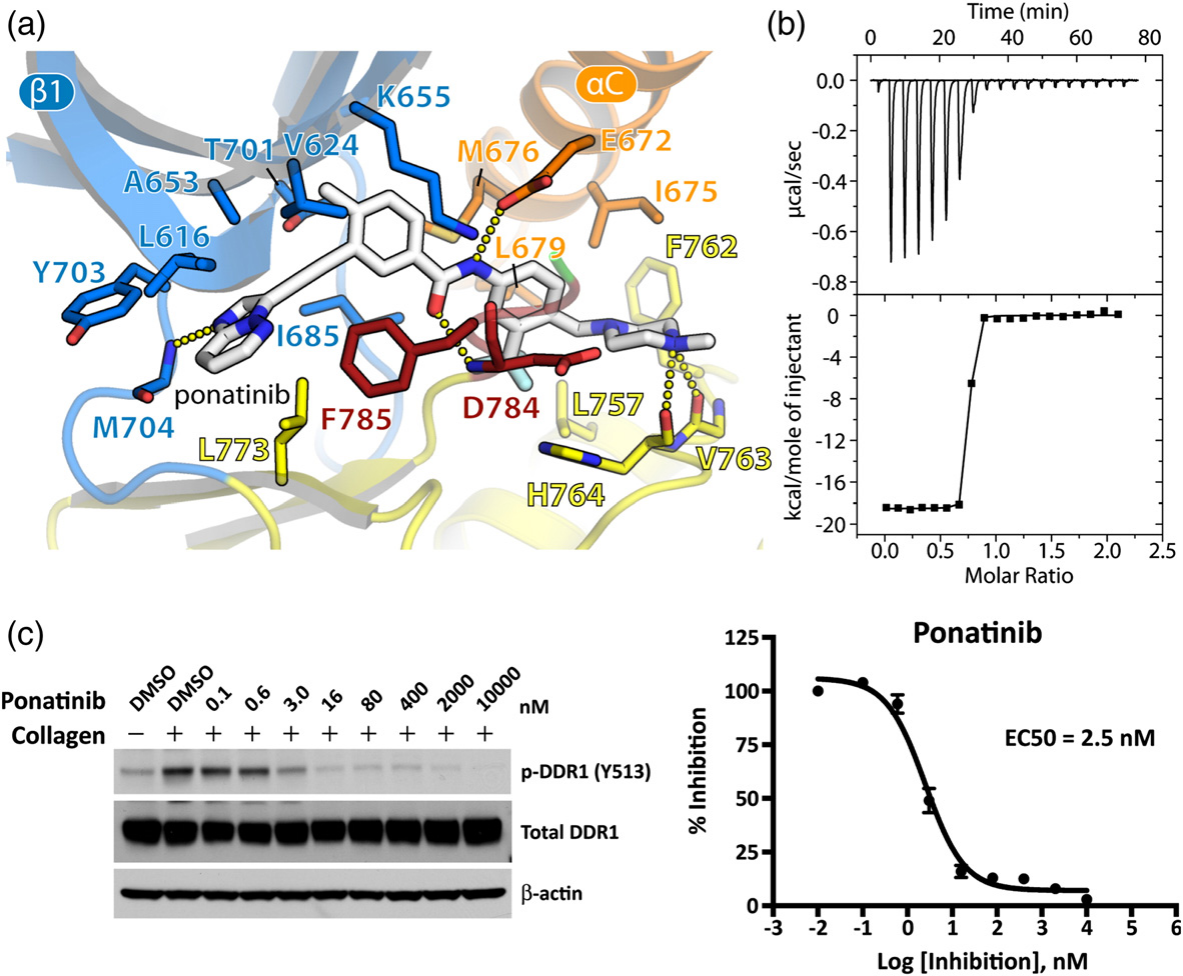
DDR1 binding and inhibition by ponatinib. (a) Interactions of ponatinib in the ATP pocket of DDR1. Colors correspond to the structural features indicated in Fig. 1. Hydrogen bond interactions are shown as dotted lines. (b) ITC measurements of the binding show a *K*_D_ value of 1.3 nM. (c) Ponatinib efficacy on blocking collagen-induced DDR1 Y513 autophosphorylation in U2OS cells.

By design, ponatinib binds at the same site with the loss of one hydrogen bond owing to the ethynyl linker, which removes any polar interaction with the gatekeeper residue Thr701 (Fig. 6a). Hinge interactions are made instead by an imidazo[1,2*b*]pyridazine head group, which establishes the hydrogen bond with Met704. In addition to the remaining four hydrogen bonds, there are extensive hydrophobic interactions across the DDR1 pocket, although the π–π interaction with Phe785 is broken (Fig. 6a). The added trifluoromethyl group occupies the pocket vacated by the inverted DFG motif and forms favorable hydrophobic contacts with Leu679, Ile684, Ile685, Leu757 and Ile782 (Fig. 6a). ITC measurements suggested that the ponatinib interaction was slightly stronger than imatinib, with a calculated *K*_D_ of 1.3 nM (Fig. 6b). Similar potency was observed in U2OS cells, where ponatinib inhibited collagen-induced DDR1 autophosphorylation with an EC_50_ of 2.5 nM (Fig. 6d).

The tight binding of both inhibitors can be understood from the similar inhibitor binding modes of DDR1 and ABL, which share a conserved threonine gatekeeper in their hinge regions (Supplementary Fig. S1). Importantly for inhibitor design, the ATP pocket of DDR1 also shows a number of sequence changes. Several substitutions cluster around the inhibitor tail region, where DDR1 Ile675 (αC), Leu679 (αC), Ile684 (αC-β4 loop) and Ile685 (αC-β4 loop) replace ABL Val289, Ile293, Leu298 and Val299, respectively. Other changes include Tyr703 (ABL Phe317) in the hinge and Met699 (ABL Ile313, β5), which packs above the methylphenyl linker.

### Differences to the binding mode of DDR1-IN-1

**Fig. 7:**
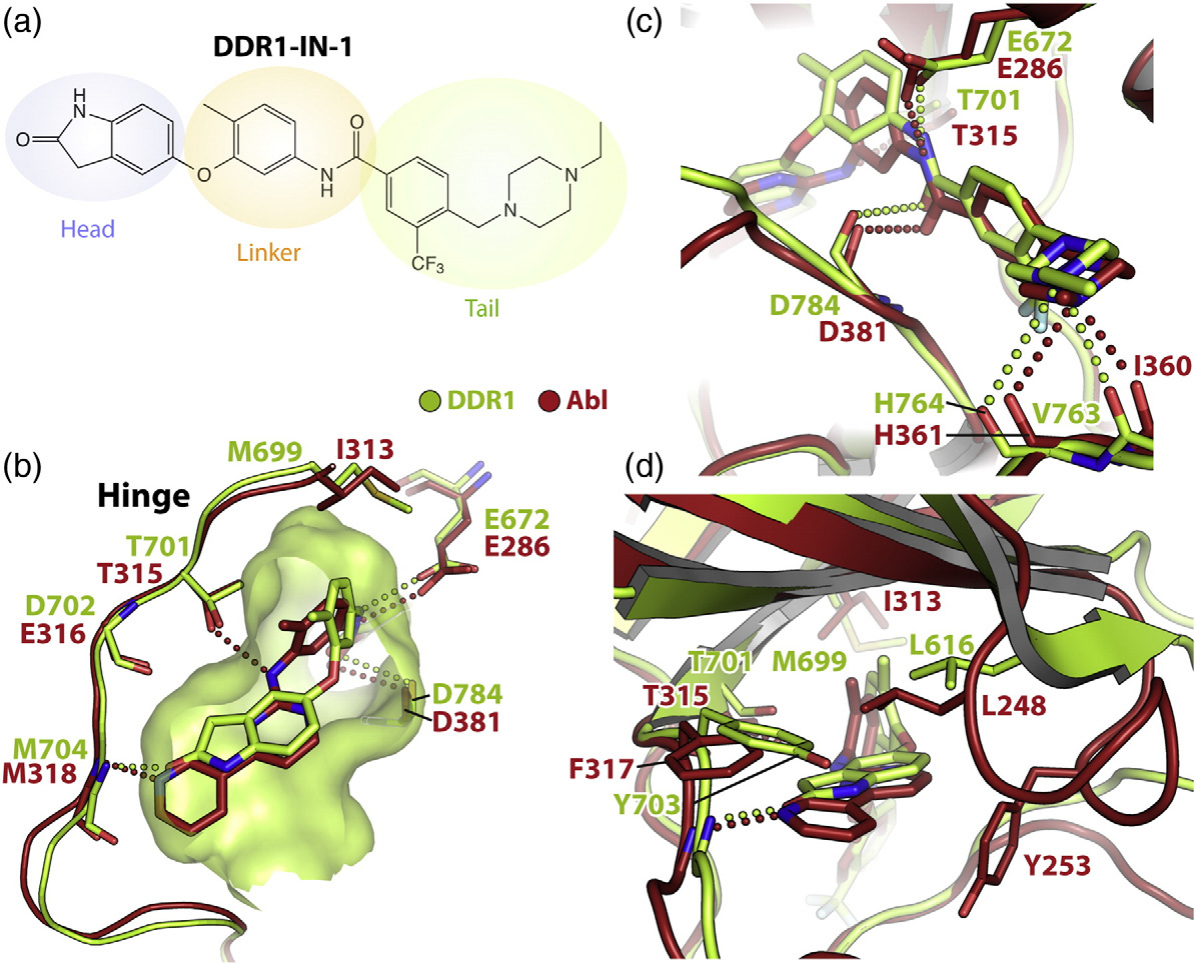
Structural basis for DDR1-IN-1 selectivity. (a) Chemical structure of DDR1-IN-1. (b) Superposition of the ABL–imatinib complex (red; PDB ID: 2HYY) and the DDR1-IN-1 complex with DDR1 (green; PDB ID: 4CKR) [35]. A green surface representation defines the shape and extent of the ATP pocket in DDR1. The alternative hinge interactions of the two inhibitors are highlighted. (c) Superposition as in (b) highlighting the interactions of the inhibitor “tail” regions. (d) The ether bridge of DDR1-IN-1 orientates the indolin-2-one “head” group away from the ABL P-loop disrupting critical hydrophobic interactions with Tyr253.

Recently we reported the discovery of a type II kinase inhibitor, DDR1-IN-1, with striking selectivity for DDR1 (Fig. 7a; PDB ID: 4CKR) [35]. This inhibitor binds the DFG-out conformation of DDR1 in a similar fashion to imatinib. The carbonyl of its indolin-2-one head group forms a hydrogen bond with Met704 in the hinge, similar to the interactions of imatinib and ponatinib (Fig. 7b). However, the ether bridge of DDR1-IN-1 eliminates the hydrogen bond to the gatekeeper residue Thr701. Similar binding interactions are also observed across the tail region of DDR1-IN-1 (Fig. 7c), where the trifluoromethyl groups of both DDR1-IN-1 and ponatinib occupy the same hydrophobic pocket created by the flip of the DFG motif.

To rationalize why DDR1-IN-1 is selective for DDR1 (IC_50_ = 105 nM) relative to ABL (IC_50_ = 1.8 μM), we modeled the DDR1 co-crystal structure (PDB ID: 4CKR) onto the ABL–imatinib complex (PDB ID: 2HYY). While the overall binding modes of the two inhibitors are predicted to be similar, one notable difference is that the ether bridge of DDR1-IN-1 adopts a different dihedral angle relative to the aniline NH of imatinib (Fig. 7b). As a result, the indolin-2-one head group of DDR1-IN-1 is orientated away from the ABL P-loop disrupting critical hydrophobic interactions with ABL Tyr253 and the hydrogen bond to the gatekeeper threonine (Fig. 7d). In addition, the hydrogen bond between Met318 in the ABL hinge and the lactam carbonyl of DDR-IN-1 is predicted to be approximately 3.0 Å, which is longer than the 2.6 Å hydrogen bond observed in the DDR1 complex. These predictions require examination of an experimental ABL structure with DDR1-IN-1.

Finally, to investigate the chemical features required in the linker and tail moieties of DDR1-IN-1, we prepared seven analogs and tested their ability alongside imatinib and ponatinib to inhibit DDR1 and DDR2 kinase activity (Table 2 and Supplementary Fig. S2) [42]. Ponatinib potently inhibited both DDR kinases with an IC_50_ of 9 nM. Inhibition by imatinib was slightly weaker with DDR1 and DDR2 exhibiting IC_50_ values of 41 and 71 nM, respectively. DDR1-IN-1 also retained significant activity against DDR1 (IC_50_ = 105 nM) despite the loss of the hydrogen bond to Thr701 and a reduction in the π–π stacking interaction that is observed between Phe785 and imatinib. We found that potency against the DDR kinases was decreased slightly when the amide between the linker and tail moieties was replaced with urea (1). Replacement of the methylpiperazine with an ether-linked methylpiperidine (2) resulted in a compound that retained the potency of DDR1-IN-1. Switching to a 3,5-substitution pattern (3,4) resulted in a slight decrease in potency relative to the 3,4-substitution pattern of DDR1-IN-1. Deletion of the methylpiperazine was tolerated when a hydrophobic 3-position substituent was maintained (5). Removal of the trifluoromethyl group resulted in analogs (6,7) that were inactive suggesting that a hydrophobic interaction near the DFG motif is likely essential for DDR1-IN-1.

## Discussion

The structures presented here were solved at high resolution and show in detail how DDR1 achieves high affinity for imatinib and ponatinib, respectively. Both type II inhibitors bind in their more potent extended conformations to the inactive DFG-out conformation of the kinase domain. Differences to ABL are observed primarily in the P-loop, where DDR1 adopts the active conformation common to the KIT–imatinib complex (KIT, mast/stem cell growth factor receptor) [43]. As a result, residues in the DDR1 P-loop that confer drug resistance when introduced in ABL are solvent exposed and tolerated. DDR1 also assembles a cage-like structure around the inhibitor pocket by tethering the activation segment to the αD helix. This alternative loop arrangement stabilizes the DFG-out conformation of DDR1 and establishes a distinct packing from other structures. This conformation is exploited by the first DDR1-selective type II inhibitors that carry variant head and linker moieties that restrict interaction with the gatekeeper residue [35,44]. Interestingly, the ether bridge of DDR1-IN-1 is also found in the MET (hepatocyte growth factor receptor) inhibitor LY2801653, which has entered clinical trials for advanced cancer and inhibits DDR1 with IC_50_ and EC_50_ values of less than 1 nM [45].

Imatinib-mediated inhibition of breakpoint cluster region-ABL has shown remarkable safety and efficacy against CML [26]. Perhaps more significantly, the recognition of imatinib activity against other kinases, notably KIT and PDGFR (platelet-derived growth factor receptor), has led to its effective use in other oncology indications [46,47] and ongoing clinical trials in fibrosis [48]. Collagen-induced activation of the RTKs DDR1 and DDR2 is similarly observed in fibrotic diseases and neoplastic tissue suggesting that DDR inhibition may be a beneficial off-target effect. Furthermore, ponatinib and dasatinib show potent activity against mutant DDR2 in models of squamous cell lung cancer [20] and indeed dasatinib has entered clinical trials for this indication [49]. DDR kinases share a conserved threonine gatekeeper residue with ABL and are therefore likely to remain susceptible to drug resistance mutations at this site. The aminopyrimidine head group of imatinib is hydrogen bonded to the gatekeeper Thr701 in DDR1 analogous to its interaction with the gatekeeper Thr315 in ABL [27]. In CML, mutation of the gatekeeper Thr315 to Ile confers drug resistance [50], suggesting that an analogous mutation in DDR1 and DDR2 would also confer resistance to imatinib.

Ponatinib overcomes drug resistance arising from the gatekeeper position but acquires reduced selectivity and increased off-target effects. Interesting in this respect is the ponatinib molecule that stabilizes the crystallographic dimer in the kinase–inhibitor complex. While we have not investigated this effect in solution, it may be of relevance for the full-length DDR1 and DDR2 receptors, which form constitutive homodimers at the cell surface. It may therefore be of interest in the future to screen this allosteric site against a library of ponatinib derivatives to identify compounds that stabilize this inactive kinase conformation.

In summary, we report the crystal structures of the human DDR1 kinase domain in complex with two clinically relevant kinase inhibitors and identify structural features that determine the binding of DDR-selective inhibitors. The high affinity of these interactions supports the potential use of these molecules to control excessive DDR signaling in diseases such as inflammation, fibrosis and lung cancer.

## Materials and Methods

### Chemicals

Unless otherwise noted, reagents and solvents were obtained from commercial suppliers and were used without further purification. For crystallization, imatinib was purchased from LC Laboratories whereas ponatinib was purchased from Selleck Chemicals. DDR1-IN-1 was prepared as described previously [35]. Synthesis of DDR1-IN-1 derivatives is described in Supplementary Material.

### Cloning

The DNA sequence corresponding to the kinase domain of human DDR1 (UniProt Q08345; residues 601–913) was cloned into the transfer vector pFB-LIC-Bse by ligation-independent cloning. The vector encodes an N-terminal hexahistidine tag and a tobacco etch virus protease A (TEV) cleavage site. Bacmid DNA was prepared from *Escherichia coli* strain DH10Bac and used to generate baculovirus in Sf9 insect cells.

### Protein expression and purification

Baculovirus was used to infect Sf9 cells grown in suspension to a density of 2 × 10^6^ cells/mL in Insect-Xpress media (Lonza). Cells were incubated at 27 °C and harvested 72 h post-infection. Harvested cells were resuspended in binding buffer [50 mM Hepes (pH 7.5), 500 mM NaCl, 5% glycerol and 5 mM imidazole] supplemented with protease inhibitor cocktail set III (Calbiochem) at 1:1000 dilution and 1 mM tris(2-carboxyethyl)phosphine (TCEP). Cells were disrupted by high-pressure homogenization. Polyethylenimine was added to a final concentration of 0.5% to precipitate DNA and the cell lysate was clarified by centrifugation at 21,000 RPM for 1 h at 4 °C.

DDR1 protein was purified using nickel-Sepharose resin (GE Healthcare) and eluted stepwise with imidazole. Following tag cleavage, we purified the protein further by size-exclusion chromatography using a HiLoad Superdex S75 26/60 column (GE Healthcare) buffered in 10 mM Hepes (pH 7.5), 250 mM NaCl, 5% glycerol and 1 mM TCEP. The eluted DDR1 protein was supplemented with 5 mM l-arginine, 5 mM l-glutamate and 2 mM dithiothreitol before concentrating for crystallization. The intact mass of the unphosphorylated protein was confirmed by electrospray ionization/time-of-flight mass spectrometry (Agilent Technologies).

### Crystallization and structure determination

Inhibitors were added to the concentrated protein in 1.5-fold molar excess and the protein solution was centrifuged at 14,000 RPM prior to crystallization. The DDR1–ponatinib complex was crystallized at 4 °C in 150 nL sitting drops mixing 100 nL protein solution at 11 mg/mL with 50 nL of a reservoir solution containing 0.1 M 2-[bis(2-hydroxyethyl)amino]-2-(hydroxymethyl)propane-1,3-diol (pH 5.5) and 25% (w/v) polyethylene glycol 3350. On mounting crystals were cryo-protected with an additional 25% ethylene glycol. Diffraction data were collected at 100 K on Diamond Light Source beamline I04-1. Crystals belonged to the monoclinic space group *P*12_1_1. Two protein molecules were present in the asymmetric unit.

The DDR1–imatinib complex was crystallized at 20 °C in 150 nL sitting drops mixing 50 nL protein solution at 8 mg/mL with 100 nL of a reservoir solution containing 0.1 M 2-[bis(2-hydroxyethyl)amino]-2-(hydroxymethyl)propane-1,3-diol (pH 6.3) and 34% (w/v) polyethylene glycol 3350. After 4 h, 20 nL of DDR1–ponatinib seed crystals was added to initiate crystal growth. On mounting crystals were cryo-protected with an additional 25% ethylene glycol. Diffraction data were collected at 100 K on Diamond Light Source beamline I03. Crystals belonged to the orthorhombic space group *P*2_1_2_1_2_1_. Two protein molecules were present in the asymmetric unit.

Data were indexed and integrated using XDS [51] and scaled using AIMLESS [52,53] in the CCP4 suite of programs [54]. Phases were found using molecular replacement in Phaser [55]. PHENIX.SCULPTOR was used to optimize PDB ID: 4AT5 (TrkB) [37] for use as a search model. The structures were built using PHENIX.AUTOBUILD [56] and then refined and modified using alternate rounds of REFMAC5 [57] and Coot [58,59]. TLS groups were determined using the TLSMD server [60]. The refined structures were validated with MolProbity [61] and the atomic coordinate files were deposited in the Protein Data Bank with Autodep [62]. Structure figures were prepared with PyMOL [63].

### Isothermal titration calorimetry

Experiments were performed at 15 °C using a Microcal VP-ITC microcalorimeter. Protein and ligands were buffered in 50 mM Hepes (pH 7.5), 250 mM NaCl, 1 mM TCEP and 2% DMSO. We titrated 100 μM DDR1 into inhibitor solutions at 10 μM concentration. Data were analyzed using a single binding site model implemented in the Origin software package provided with the instrument.

### IC_50_ determination

IC_50_ values were determined by Invitrogen using a LanthaScreen kinase activity assay.

### EC_50_ determination

U2OS cells containing tetracycline-inducible human HA-FLAG-DDR1 expression were used for the EC_50_ test. DDR1 was induced by 2 μg/mL doxycycline for 48 h prior to DDR1 activation by rat tail collagen I. The cells were pre-treated by media containing each concentration of the compound for 1 h and treated by changing the media to the EC_50_ test media containing 10 μg/mL collagen and each concentration of the compound for 2 h. Cells were washed with cold phosphate-buffered saline three times and lysed with lysis buffer [20 mM Tris (pH 7.5), 5 mM ethylenediaminetetraacetic acid, 1% Triton X-100, protease inhibitor cocktail and phosphatase inhibitor cocktail]. The phosphorylation of DDR1 and total DDR1 expression were quantified by using the ImageJ program following Western blot using anti-human DDR1b (Y513) and anti-HA, respectively. β-Actin was blotted as loading control. The EC_50_ concentration was calculated by nonlinear regression analysis using GraphPad Prism software.

### Protein Data Bank accession numbers

Atomic coordinates and structure factors have been deposited in the Protein Data Bank under accession numbers 3ZOS and 4BKJ3ZOS4BKJ.

## Figures and Tables

**Table 1:** Data collection and refinement statistics.

	DDR1–ponatinib (3ZOS)	DDR1–imatinib (4BKJ)
*Data collection*		
X-ray source	Diamond Light Source I04-1	Diamond Light Source I03
Wavelength (Å)	0.92	0.9795
Resolution range (Å)^a^	45.41–1.92 (1.988–1.919)	45.14–1.7 (1.76–1.699)
Space group	*P*12_1_1	*P*2_1_2_1_2_1_
Unit cell (Å)		
*a*	69	60
*b*	61.7	60
*c*	80.2	180.6
Unit cell (°)		
α	90	90
β	104.4	90
γ	90	90
Unique reflections^a^	49,502 (3453)	72,847 (4093)
Multiplicity^a^	6.1 (6.1)	6.5 (6.5)
Completeness (%)^a^	99 (98.7)	99.9 (99.7)
*I*/σ(*I*)^a^	9.1 (2.7)	13.5 (2.9)
*R*_merge_^a^	0.137 (0.785)	0.08 (0.632)
*Refinement*		
*R*_work_	0.206	0.157
*R*_free_	0.234	0.176
Number of atoms	5224	5175
RMSD bonds (Å)	0.012	0.007
RMSD angles (°)	1.49	1.23
Average *B*-factor (Å^2^)	23.8	24.6
Average *B*-factor macromolecules (Å^2^)	23.7	24.3
Average *B*-factor ligands (Å^2^)	19.8	21.8
Average *B*-factor solvent (Å^2^)	26.4	30.6

a Values in parentheses refer to the highest-resolution shell.

**Table 2:** Structure–activity relationship of DDR1-IN-1.


Compounds	R	Enzymatic IC_50_ values (nM)	
		DDR1	DDR2
Ponatinib		**9.4**	**9.0**
Imatinib		**41**	**71.6**
DDR-IN-1		**105**	**413**
**1**		**262**	**785**
**2**		**115**	**338**
**3**		**182**	**546**
**4**		**150**	**385**
**5**		**174**	**1020**
**6**		**> 10,000**	**> 10,000**
**7**		**8940**	**> 10,000**
