# Supplementary material for: Structural Mechanisms Determining Inhibition of the Collagen Receptor DDR1 by Selective and Multi-Targeted Type II Kinase Inhibitors
Source: J Mol Biol. 2014 Jun 26;426(13):2457–70. doi: 10.1016/j.jmb.2014.04.014 (PMC4058747; doi:10.1016/j.jmb.2014.04.014)
Supplement: Supplementary file 1 — Supplementary material. [file mmc1.docx]

**Supplementary Material**

**Structural mechanisms determining inhibition of the collagen receptor DDR1 by selective and multi-targeted type II kinase inhibitors**

Peter Canning^1^, Li Tan^2,3^, Kiki Chu^4^, Sam W. Lee^4^, Nathanael S. Gray^2,3^* and Alex N. Bullock^1^*

1. Structural Genomics Consortium, University of Oxford, Old Road Campus, Roosevelt Drive, Oxford OX3 7DQ, UK
2. Department of Cancer Biology, Dana Farber Cancer Institute, Boston, MA, 02115, USA
3. Department of Biological Chemistry and Molecular Pharmacology, Harvard Medical School, Boston, MA, 02115, USA
4. Cutaneous Biology Research Center, Massachusetts General Hospital and Harvard Medical School, Charlestown, Massachusetts 02129, USA

**Chemical synthesis and spectral data**

Unless otherwise noted, reagents and solvents were obtained from commercial suppliers and were used without further purification. ^1^H NMR spectra were recorded on 600 MHz (Varian AS600), and chemical shifts are reported in parts per million (ppm, δ) downfield from tetramethylsilane (TMS). Coupling constants (*J*) are reported in Hz. Spin multiplicities are described as s (singlet), brs (broad singlet), t (triplet), q (quartet), and m (multiplet). Mass spectra were obtained on a Waters Micromass ZQ instrument. Preparative HPLC was performed on a Waters Symmetry C18 column (19 x 50 mm, 5µM) using a gradient of 15-95% methanol in water containing 0.05% trifluoacetic acid (TFA) over 22 min (28 min run time) at a flow rate of 20 mL/min. Purities of assayed compounds were in all cases greater than 95%, as determined by reverse-phase HPLC analysis.

**methyl 2-(5-(2-methyl-5-nitrophenoxy)-2-nitrophenyl)acetate (8).** To a stirred solution of methyl 2-(5-fluoro-2-nitrophenyl)acetate (790 mg, 3 mmol) and 2-methyl-5-nitrophenol (700 mg, 4.5 mmol) in 2 mL of DMSO was added K_2_CO_3_ (830 mg, 6 mmol). The reaction mixture was allowed to stand for 1 hr at 100 ^o^C, and then cooled to RT. The mixture was acidified with 1N HCl solution and extracted with ethyl acetate, the organic phase was washed with water and brine, dried over sodium sulfate, concentrated and purified with column chromatography (hexane : ethyl acetate 3:1). 830 mg (80%) of **8** was obtained. ^1^H NMR (600 MHz, CDCl_3_) δ 8.18 (d, *J* = 8.4 Hz, 1H), 8.05 (dd, *J* = 8.4, 2.4 Hz, 1H), 7.85 (d, *J* = 2.4 Hz, 1H), 7.48 (d, *J* = 9.0 Hz, 1H), 6.91 (dd, *J* = 9.6, 2.4, 1H), 6.86 (d, *J* = 2.4, 1H), 3.99 (s, 2H), 3.71 (s, 3H), 2.32 (s, 3H).

**5-(5-amino-2-methylphenoxy)indolin-2-one** **(9).** To a stirred solution of compound **8** (690 mg, 2 mmol) in 30 mL of ethyl acetate and 3 mL of ethanol was added tin chloride dihydrate (2.26 g, 10 mmol). The reaction mixture was allowed to stand for 4~5 hr at 80 ^o^C, and then cooled to RT. The mixture was diluted with ethyl acetate and added saturated sodium bicarbonate solution, and stirred for 15 min, the organic phase was separated and washed with water and brine, concentrated and purified with column chromatography (dichloromethane : methanol 15:1). 380 mg (75%) of **9** was obtained. ^1^H NMR (600 MHz, CD_3_OD) δ 6.97 (d, *J* = 8.4 Hz, 1H), 6.87 (d, *J* = 2.4 Hz, 1H), 6.83 (d, *J* = 8.4 Hz, 1H), 6.78 (dd, *J* = 9.0, 2.4 Hz, 1H), 6.44 (dd, *J* = 7.8, 2.4, 1H), 6.24 (d, *J* = 2.4, 1H), 3.50 (s, 2H), 2.09 (s, 3H). MS (ESI) *m/z* 255 (M+H)^+^.

***N*-(4-methyl-3-((2-oxoindolin-5-yl)oxy)phenyl)-4-((1-methylpiperidin-4-yl)oxy)-3-(trifluoromethyl)benzamide (2).** To a solution of compound **9** (51 mg, 0.2 mmol), **10** (73 mg, 0.24 mmol) and HATU (91 mg, 0.24 mmol) in 3 mL of dichloromethane was added DMAP (30 mg, 0.24 mmol) and DIEA (87 µL, 0.5 mmol) and the reaction mixture was stirred overnight. The mixture was concentrated and purified with HPLC. 119 mg (76%) of **2** was obtained as white solid**.** ^1^H NMR (600 MHz, TFA salt, DMSO) δ 10.32 (s, 1H), 10.21 (s, 1H), 8.20 (s, 1H), 8.18 (s, 1H), 7.48 (m, 1H), 7.45 (d, *J* = 8.4, 1H), 7.28 (s, 1H), 7.24 (d, *J* = 9.0 Hz, 1H), 6.89 (m, 2H), 3.43-3.51 (m, 5H), 3.47 (s, 2H), 3.10 (s, 2H), 2.91 (m, 2H), 2.82 (s, 3H), 2.30 (s, 3H). MS (ESI) *m/z* 540 (M+H)^+^.

**1-(4-((4-ethylpiperazin-1-yl)methyl)-3-(trifluoromethyl)phenyl)-3-(4-methyl-3-((2-oxoindolin-5-yl)oxy)phenyl)urea (1).** To a stirred solution of aniline **11** (144 mg, 0.5 mmol) in 3 mL of tetrahydrofuran was added triphosgene (50 mg, 0.17 mmol) and followed by triethylamine (70 µL, 0.5 mmoL) dropwise at 0 ^o^C. The reaction mixture was allowed to warm to RT slowly and stirred overnight, then was added intermediate **9** (127 mg, 0.5 mmol) and triethylamine (140 µL, 1.0 mmoL). The result mixture was allowed to stand for 24 hr at 75 ^o^C, and then cooled to RT, then diluted with ethyl acetate, the organic phase was separated and washed with water and brine, concentrated and purified with column chromatography (dichloromethane : methanol 15:1). 200 mg (71%) of **1** was obtained as pale yellow solid. ^1^H NMR (600 MHz, DMSO) δ 10.30 (s, 1H), 8.81 (s, 1H), 8.70 (s, 1H), 8.18 (s, 1H), 7.60 (m, 1H), 7.56 (s, 1H), 7.49 (d, *J* = 9.0 Hz, 1H), 7.15 (d, *J* = 9.0 Hz, 1H), 7.07 (d, *J* = 8.4 Hz, 1H), 6.97 (s, 1H), 6.84 (s, 1H), 6.77 (s, 1H), 3.48 (s, 2H), 3.45 (s, 2H), 3.10 (s, 2H), 2.16-2.47 (m, 12H), 2.12 (s, 3H), 0.95 (m, 3H). MS (ESI) *m/z* 568 (M+H)^+^.

**3-(4-(2-hydroxyethyl)piperazin-1-yl)-*N*-(4-methyl-3-((2-oxoindolin-5-yl)oxy)phenyl)-5-(trifluoromethyl)benzamide (3).** Prepared with same method as compound **2**, yield 70% from **9**. ^1^H NMR (600 MHz, TFA salt, DMSO) δ 10.32 (s, 1H), 10.25 (s, 1H), 8.19 (m, 1H), 7.56 (m, 1H), 7.44 (d, *J* = 8.4 Hz, 1H), 7.36 (m, 1H), 7.27 (s 1H), 7.24 (d, *J* = 8.4 Hz, 1H), 6.88 (s, 1H), 6.79 (m, 2H), 3.60 (m, 4H), 3.45 (s, 2H), 3.25 (m, 4H), 3.12 (m, 2H), 2.35-2.70 (m, 2H), 2.18 (s, 3H). MS (ESI) *m/z* 555 (M+H)^+^.

**3-(4-ethylpiperazin-1-yl)-*N*-(4-methyl-3-((2-oxoindolin-5-yl)oxy)phenyl)-5-(trifluoromethyl)benzamide (4).** Prepared with same method as compound **2**, yield 79% from **9**. ^1^H NMR (600 MHz, TFA salt, DMSO) δ 10.33 (s, 1H), 10.29 (s, 1H), 9.63 (m, 1H), 7.68 (s, 1H), 7.64 (s, 1H), 7.46 (s, 1H), 7.44 (d, *J* = 8.4 Hz, 1H), 7.27 (s, 1H), 7.25 (d, *J* = 9.0 Hz, 1H), 6.88 (s, 1H), 6.80 (m, 2H), 4.07 (m, 2H), 3.57 (m, 2H), 3.46 (s, 2H), 3.19 (m, 2H), 3.08 (m, 2H), 2.18 (s, 3H), 1.24 (t, *J* = 7.2 Hz, 1H). MS (ESI) *m/z* 539 (M+H)^+^.

**3-(2-cyanopropan-2-yl)-*N*-(4-methyl-3-((2-oxoindolin-5-yl)oxy)phenyl)benzamide (5).** Prepared with same method as compound **2**, yield 83% from **9**. ^1^H NMR (600 MHz, TFA salt, DMSO) δ 10.31 (s, 1H), 10.21 (s, 1H), 7.95 (s, 1H), 7.86 (d, *J* = 2.4 Hz, 1H), 7.70 (d, *J* = 2.4 Hz, 1H), 7.54 (t, *J* = 7.5 Hz, 1H), 7.45 (d, *J* = 2.4 Hz, 1H), 7.29 (s, 1H), 7.24 (d, *J* = 7.8 Hz, 1H), 6.88 (s, 1H), 6.79 (m, 2H), 3.45 (s, 2H), 2.18 (s, 3H), 1.70 (s, 6H). MS (ESI) *m/z* 426 (M+H)^+^.

**4-((4-ethylpiperazin-1-yl)methyl)-2-fluoro-*N*-(4-methyl-3-((2-oxoindolin-5-yl)oxy)phenyl)benzamide (6).** Prepared with same method as compound **2**, yield 81% from **9**. ^1^H NMR (600 MHz, TFA salt, DMSO) δ 10.32 (s, 1H), 10.28 (s, 1H), 7.57 (t, *J* = 7.8 Hz, 1H), 7.38 (d, *J* = 8.4 Hz, 1H), 7.19-7.28 (m, 2H), 7.24 (s, 1H), 7.21 (s, 1H), 6.87 (s, 1H), 6.78 (m, 2H), 3.66 (s, 2H), 3.45 (s, 2H), 3.42 (m, 2H), 3.10 (m, 2H), 2.85-3.04 (m, 4H), 2.37 (m, 3H), 2.17 (s, 3H), 1.18 (t, *J* = 7.2 Hz, 3H). MS (ESI) *m/z* 503 (M+H)^+^.

**5-((4-ethylpiperazin-1-yl)methyl)-2-fluoro-*N*-(4-methyl-3-((2-oxoindolin-5-yl)oxy)phenyl)benzamide (7).** Prepared with same method as compound **2**, yield 79% from **9**. ^1^H NMR (600 MHz, DMSO) δ 10.30 (s, 1H), 10.29 (s, 1H), 7.45 (d, *J* = 7.2 Hz, 1H), 7.42 (m, 1H), 7.38 (d, *J* = 8.4 Hz, 1H), 7.22 (m, 2H), 7.21 (s, 1H), 6.87 (s, 1H), 6.78 (m, 2H), 3.45 (s, 2H), 3.43 (s, 2H), 2.20-2.44 (m, 8H), 2.27 (q, *J* = 7.2 Hz, 2H), 2.17 (s, 3H), 0.94 (t, *J* = 7.2 Hz, 3H). MS (ESI) *m/z* 503 (M+H)^+^.


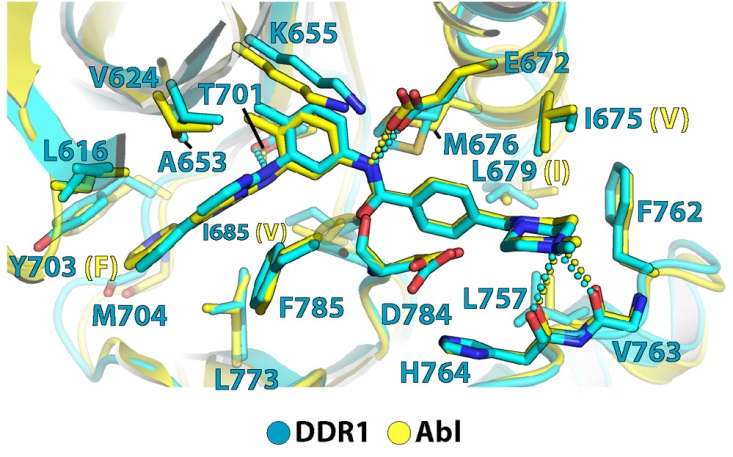


**Figure S1. Comparison of imatinib binding to DDR1 and ABL.** Superposition of the DDR1-imatinib (blue) and ABL-imatinib (PDB 2HYY, yellow)^40^ complexes reveals their similar binding modes. Residue numbering is shown for DDR1; Substitutions in ABL are shown in parentheses.


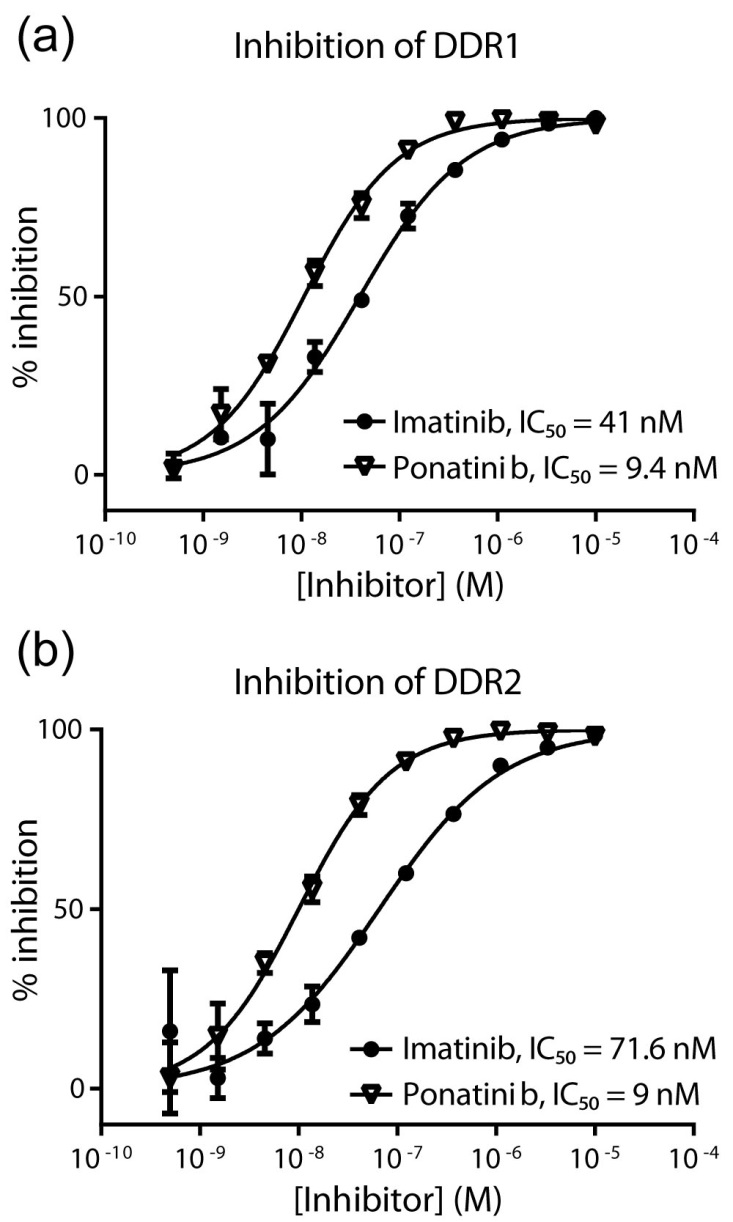


**Figure S2. Inhibition of DDR1 and DDR2. (a)** IC_50_ values for DDR1 inhibition determined by using a LanthaScreen kinase activity assay (Invitrogen). **(b)** IC_50_ values for DDR2 inhibition determined similarly.
